# Human infection challenge in the pandemic era and beyond, HIC-Vac annual meeting report, 2022

**DOI:** 10.1093/immadv/ltad024

**Published:** 2023-10-31

**Authors:** Megan V C Barnes, Anika Mandla, Emma Smith, Maija Maskuniitty, Peter J M Openshaw, HIC-Vac meeting contributors, Yara-Natalie Abo, Stephanie Ascough, Helen Ashwin, Panisadee Avirutnan, Andrew P Catchpole, Primus Che Chi, Christopher Chiu, Thomas C Darton, Emmanuella Driciru, Dingase Dula, Daniela M Ferreira, Alastair Fraser, Phoebe Garrett, Diane Gbesemete, Stephen Gordon, David L Heymann, Emma Houlder, Euzebiusz Jamrozik, Melissa Kapulu, Enock Kessy, Anna M Overgaard Kildemoes, Jan Pieter Koopman, Helen McShane, Oranich Navanukroh, Faith H Osier, Joshua Osowicki, Vy Pham, Andrew J Pollard, Woraphat Ratta-apha, Sarah E Silk, Saranya Sridhar, Kena A Swanson, Kawsar R Talaat, Ryan S Thwaites, Orly Welch

**Affiliations:** National Heart and Lung Institute, Imperial College London, London, UK; National Heart and Lung Institute, Imperial College London, London, UK; Department of Infectious Disease, Imperial College London, London, UK; National Heart and Lung Institute, Imperial College London, London, UK; National Heart and Lung Institute, Imperial College London, London, UK; Murdoch Children’s Research Institute, the Royal Children’s Hospital Melbourne, Australia; Imperial College London, UK; University of York, UK; Mahidol University, Thailand; hVIVO, UK; KEMRI Wellcome Research Programme, Kenya; Imperial College London, UK; University of Sheffield, UK; CHI-S-Uganda, Leiden University Medical Center, the Netherlands; Malawi–Liverpool–Wellcome Trust Clinical Research Programme, Malawi; University of Oxford, UK, and Liverpool School of Tropical Medicine, UK; 1Day Sooner, UK; 1Day Sooner, UK; University of Southampton, UK; Malawi–Liverpool–Wellcome Trust Clinical Research Programme, Malawi; London School of Hygiene and Tropical Medicine, UK; Leiden University Medical Center, the Netherlands; University of Oxford, UK; KEMRI Wellcome Research Programme, Kenya; Ifakara Health Institute, Tanzania; Leiden University Medical Center, the Netherlands; Leiden University Medical Center, the Netherlands; University of Oxford, UK; Mahidol University, Thailand; Imperial College London, UK; Murdoch Children’s Research Institute, the Royal Children’s Hospital Melbourne, and the University of Melbourne, Australia; Oxford University Clinical Research Unit, Vietnam; University of Oxford, UK; Mahidol University, Thailand; University of Oxford, UK; Sanofi Pasteur and Inno4Vac, UK; Pfizer, USA; Johns Hopkins University, USA; Imperial College London, UK; 1Day Sooner, UK

**Keywords:** controlled human infection models, mucosal immunology, low-middle-income countries, infectious disease, vaccination, experimental medicine

## Abstract

HIC-Vac is an international network of researchers dedicated to developing human infection challenge studies to accelerate vaccine development against pathogens of high global impact. The HIC-Vac Annual Meeting (3rd and 4th November 2022) brought together stakeholders including researchers, ethicists, volunteers, policymakers, industry partners, and funders with a strong representation from low- and middle-income countries. The network enables sharing of research findings, especially in endemic regions. Discussions included pandemic preparedness and the role of human challenge to accelerate vaccine development during outbreak, with industry speakers emphasising the great utility of human challenge in vaccine development. Public consent, engagement, and participation in human challenge studies were addressed, along with the role of embedded social science and empirical studies to uncover social, ethical, and regulatory issues around human infection challenge studies. Study volunteers shared their experiences and motivations for participating in studies. This report summarises completed and ongoing human challenge studies across a variety of pathogens and demographics, and addresses other key issues discussed at the meeting.

## Introduction

**Figure 1:**
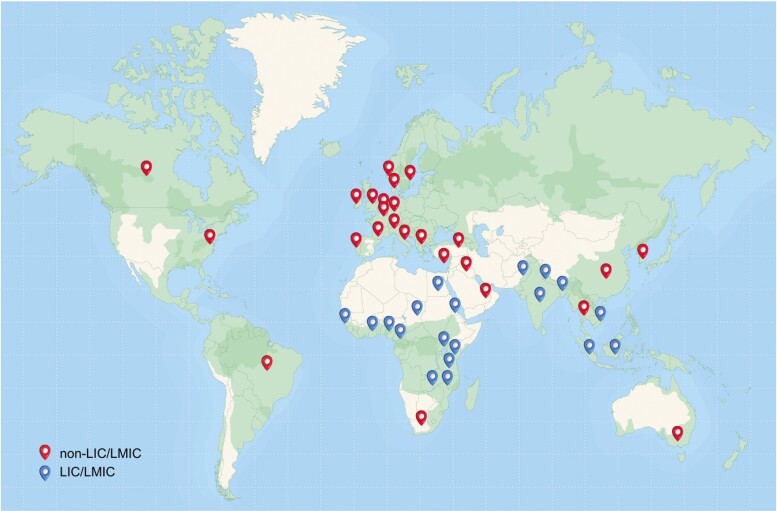
Membership map of countries comprising current HIC-Vac members. Red pins represent non-LMIC and blue pins represent LIC/LMIC. LIC: low income countries; LMIC: low-middle income countries. Figure produced by M.Maskuniitty.

Established in July 2017 with MRC/BBSRC funding, and currently funded by the Wellcome Trust, the HIC-Vac network aims to support, develop, and encourage the use of human infection challenge (HIC) studies (also referred to as controlled human infection model (CHIM) studies), for pathogens causing high global disease burden. The UK is a world leader in HIC studies with a concentration of unique expertise, a strong history of HIC and a relatively supportive legal, regulatory, and ethical environment. HIC-Vac draws together our collective experience and shares best practice, supporting the development of new research initiatives in testing vaccine safety and efficacy. The network is led by Peter Openshaw (Director) and Andrew J Pollard (Co-director) from Imperial College London and University of Oxford respectively. As of late 2023, there are 471 members, including 152 from low/low-and-middle-income countries (LIC/LMIC; Fig. 1). HIC-Vac has provided seed funding to projects contributing to larger award applications or to extend existing sample analysis and training/public involvement awards. The network hosts annual meetings to build the community, advance the field of human infection studies, and improve public health outcomes globally. The 2022 annual meeting was held near Leatherhead (UK) on the 3rd and 4th November 2022. Seventy-four delegates attended in person, with others joining remotely. Here, we discuss the key findings presented at the meeting, including some unpublished data, as well as key considerations for HIC studies from volunteer perspectives. As this report focuses on work presented at the 2022 HIC-VAC meeting, we acknowledge the other important HIC studies discussed at previous HIC-Vac meetings (such as the BCG challenge model for *Mycobacterium Tuberculosis* [1]; the hookworm controlled human infection study [2]; experimental human infection with *Neisseria Gonorrhoeaea* [3, 4]; the zika virus human challenge model [5]; and dengue human infection models [6]) are not discussed in this report.

## HIC studies using bacterial challenge strains

### *Streptococcal* and *Staphylococcal* challenge models

Skin infections with *Streptococcus pyogenes* (including Group A Streptococcal (GAS) disease) or *Staphylococcus aureus* are common, particularly in LMICs and at the extremes of age, with infection causing impetigo, cellulitis, and necrotising fasciitis [7]. Antibiotic therapy is generally effective, but the development of vaccines is prioritised because of increasing antibiotic resistance [8]. Post-infectious glomerulonephritis, rheumatic fever, and rheumatic heart disease continue to cause long-term illness in some settings. As animal models do not accurately represent human skin disease, skin immunology requires more research using human studies. Recent advancements have been made in this field: challenge strains, such as M75 611024 for GAS [9] and cc45 CHAL3 for *S. aureus*, have been developed. New tools e.g. full-length 16S sequencing have been introduced, allowing for a wider range of data collection during infection studies and accurate characterisation of skin bacteria before and during challenge. The use of a microneedle derma roller has also been implemented to ensure an efficient “take” of the challenge strain with minimal skin abrasion. Dose-finding studies for *S. pyogenes* pharyngitis are being performed safely [7] (University of Melbourne, Murdoch Children’s Research Institute and the Royal Children’s Hospital, Melbourne), and punch biopsies used in BCG studies provide insights into tissue collection [10].

Pneumococcal challenge studies using *Streptococcus pneumoniae* [Liverpool School of Tropical Medicine (LSTM; UK), and Malawi Liverpool Wellcome Programme (Blantyre, Malawi)] have demonstrated safety and value; however, when transferred to an endemic country e.g. Malawi, the host–pathogen interactions appear to be different [11]. In Malawi, following the introduction of the Pneumococcal conjugate vaccine (PCV13), high persistence of vaccine-type pneumococci was observed in vaccinated children, indicating poor herd immunity. Additionally, high pneumococcal carriage rates were also observed in HIV-infected populations [11]. A feasibility study in Malawi demonstrated successful colonisation with the challenge strain, but non-experimental strains remained prevalent [12]. A study comparing PCV13 efficacy in Malawian adults with those in Liverpool suggested similar protection levels (~60–70%), which raised questions about high natural carriage rates and lower experimental carriage rates in the community [13]. Future studies aim to characterise infections in special populations, test vaccine candidates and develop new challenge strains [13].

During the COVID-19 pandemic, hospitalizations from pneumococcal disease in Israel declined despite stable pneumococcal carriage rates, implicating respiratory co-infections as a cause of conversion from carriage to pathogenicity [14]. Findings from co-infection HIC studies suggest that prior influenza challenge increases the chances of pneumococcal colonisation and severity of bacterial infection, while pneumococcal challenge prior to SARS-CoV-2 reduces antiviral immune responses, indicating the importance of the order of infections [15–17]. Finally, challenge studies using Serotype 3 (SPN3), which causes invasive pneumococcal disease, have been conducted in healthy adults, with different attack rates observed for different doses and clades [18]. This study identified a suitable challenge dose and the model is now being used to evaluate vaccine efficacy against SPN3 [19].

### Shigella

Shigella is a major cause of Shigellosis: a diarrhoeal disease responsible for a high number of deaths worldwide, making it a priority for the World Health Organisations’ (WHO) vaccine development efforts [20]. Four species of Shigella (*S. dysenteriae*, *S. flexneri*, *S. boydii* and *S. sonnei*), have distinct serotypes, posing challenges such as serotype diversity (>50 altogether), lack of reliable protection indicators, and varying immunogenicity across countries and age groups [21, 22]. Numerous Shigella vaccines are in development, including: including Flexyn2a (LimmaTech Biologics) [23–25], altSonflex-1-2-3 (GSK) [26], and SF2a-TT15 (Pasteur Institute) [27]. HIC studies have assessed these vaccines, indicating immune response variations based on the route of administration and challenge species [28].

Shigella challenge models have been translated to endemic populations, with ongoing seroepidemiological studies in Kenyan adults to understand exposure and natural immunity to different strains. Extensive community engagement, involving ethical and regulatory committees, young populations, and community representatives, has led to HIC studies being included in Kenya’s national guidelines. Implementation of the well-characterised *S. sonnei* 53G model with a lyophilised challenge strain is underway, with plans for a dose-escalation study to determine the optimal attack rate based on past exposure [29, 30].

### Neisseria lactamica

*Neisseria lactamica* (*N. lactamica*), a non-pathogenic relative of *Neisseria meningitidis*, shows promise in limiting meningococcal colonisation [31, 32]. Nasal inoculation achieves colonisation rates of 33–100% in challenge studies, dependent on dose, potentially preventing acquisition and displacement of meningococcal carriage. In the meningitis belt of sub-Saharan Africa, MenA vaccine reduced cases [33], but other strains are emerging. A challenge model in Bamako, Mali [University of Oxford (UK), University College London (UK), and University of Maryland (USA)] explores *N. lactamica*’s potential to limit colonisation [34]. A further dose-finding study at the University of Southampton (UK) achieved high colonisation rates, correlating with increased *N. lactamica* IgG levels. Future studies will investigate impacts on meningococcal carriage and disease [35, 36].

## Respiratory virus HIC studies investigating mucosal immune responses

### SARS-CoV-2

The COVID-19 human challenge study [COVHIC001; Imperial College London (UK)] provided valuable insights into mucosal immune responses to SARS-CoV-2 compared to serum responses, showing dynamic mucosal cytokine production and robust responses in infected individuals that had no previous COVID-19 infection [37]. Key findings showed that mucosal immune responses were not prominent during the viral incubation period, IFN-γ suppressed viral replication, and antibody responses facilitated viral elimination. It was also indicated that pre-existing antibodies may be associated with delayed infection kinetics [38]. Additionally, samples collected through the ISARIC4C consortium (UK-wide collaboration) allowed comparisons of mucosal responses to SARS-CoV-2 across disease severities of hospitalised patients, revealing significant differences in early IFN-γ response and immune profiles.

Data was also presented from the ongoing SARS-CoV-2 human challenge study in seropositive people (COVCHIM, University of Oxford, clinicaltrials.gov NCT04864548 [39]). Dose escalation up to a dose of 105TCID50 was undertaken, with the aim of achieving a 50% infection rate.

## Respiratory syncytial virus

The INFLAMMAGE study (Imperial College London; UK) focused on assessing immune responses to respiratory syncytial virus (RSV) infection in older individuals using human challenge [40]. The study observed a high attack rate (77%) in older challenge participants, with symptoms, which were largely confined to the upper respiratory tract, peaking on day seven. Viral loads peaked on day six and were higher in the older age groups compared to younger volunteers. In younger adults, serum neutralising antibody titres and levels of IgG specific for virus-derived fusion (F) protein are weak correlates of protection against RSV. In older adults it was observed that IgG, neutralising antibodies, and antibody-secreting B cells all responded robustly to RSV infection, showing no age-related decline. In contrast to this, the secretory F-protein-specific IgA titres, previously identified as the best correlate of protection in younger adults, did not increase during infection in older adults. It did not appear to confer any protection against infection in this age group, unlike in the serum neutralising titre, which was more predictive of protection. Therefore, vaccines that boost systemic responses, but not mucosal responses, might be more effective in older adults.

## Established and developing HIC models of parasitic infections

### Plasmodium falciparum

Malaria, caused by the parasite *Plasmodium falciparum*, remains a significant health challenge, but the newly approved RTS,S/AS01 vaccine shows promise [41]. Other vaccines, including R21/Matrix-M, are in trials [42], but additional vaccines are likely to be needed. Passive transfer experiments have shown potential in blocking merozoite binding and red blood cell invasion, but blocking merozoite binding may be too broad of a target [43]. One approach to assist vaccine development uses the “KILchip” microarray, which enables detection of antibodies associated with assessing potential vaccine antigens, leading to prioritisation of merozoite-stage antigens.

Human challenge with malaria has been performed by the Jenner Institute (Oxford, UK) in collaboration with others. This enables testing of pre-erythrocytic vaccines, blood-stage vaccines, and transmission-blocking vaccines [44]. HIC models have also been established in various parts of Kenya (sponsored by the University of Oxford), where there is no active/existing malaria exposure (such as Nairobi), but also in parts of Kenya with existing malaria exposure. The aim of this was to identify what happens when the model was moved to an endemic area with participants with pre-existing immunity and high levels of exposure (the CHMI-SIKA study, clinicaltrials.gov NCT02739763) [45]. A range of different patterns of parasite growth were seen in endemic areas that differ from the typical growth pattern seen in Nairobi/high-income countries [46]. The study findings emphasised the importance of background immunity in infection outcomes. To continue this research further, a transmission challenge model based on sporozoite administration is being implemented to evaluate interventions. A vaccine efficacy study is also underway to assess the protective efficacy of different vaccine candidates, in collaboration with the Jenner Institute (Oxford, UK) [47].

In Tanzania, a phase II trial facility for HIC studies has been established with a collaboration between Ifakara Health Institute (Tanzania) and University of Oxford (UK) [48]. This capacity enables vaccine down-selection for clinical trials and ensures relevant population studies. The blood-stage controlled human malaria infection model (CHMI) was established at the facility for the first time (clinicaltrials.gov NCT04788862 [49]). Differences in parasite growth dynamics have been observed between high and low malaria pre-exposed participants, reiterating the importance of conducting studies in malaria endemic settings and the impact of existing immunity. No safety concerns were raised, parasite numbers were monitored by twice daily qPCR and the participants were all successfully treated. There was good concordance between the data generated in Tanzania and in Oxford, with a higher parasitaemia threshold for diagnosis in Tanzanian participants without displaying symptoms. New parasite growth rate models are needed to study the differences in the growth dynamics observed in Tanzania. Now the blood-stage CHMI model is established, future HIC studies can be conducted for vaccine down selection as well as investigation of higher parasite doses, examining gametocyte development and transmission to mosquitoes.

### Leishmania

A HIC model for the single-celled parasite Leishmania, responsible for fatal visceral leishmaniasis, is being developed (Leish Challenge project, University of York, UK, Charles University, Prague), The Hebrew University of Jerusalem, Israel). The development of a HIC model for Leishmania could accelerate vaccine development and triaging, as well as providing more information about pathogenesis [50]. There are three vaccines in development, with one, ChAd63-KH, currently undergoing a phase II clinical trial [51, 52], while the other two are in pre-clinical stages. Following the screening of 28 potential participants, 14 biting visits were organised. Three cases experienced biting failure, while 11 bites resulted in lesions. Out of these, 10 lesions were suitable for biopsy, yielding valuable insights into the distribution of immune cells and parasites within the lesion using conventional pathology and spatial transcriptomics. Future work will involve further characterization of the lesions, including single-cell RNA sequencing.

### Schistosoma mansoni

Schistosomiasis, the disease caused by *Schistosoma mansoni (S. mansoni)*, poses a significant public health challenge in Uganda, with over half of the population at risk of infection and a prevalence rate of 25.6%. Most of the pathology linked to schistosomiasis is a result of immune responses to parasite eggs. As a result, to conduct HIC studies, parasites of a single-sex are used to prevent mating and egg production. At the Leiden University Medical Center (the Netherlands), a model has been established that relies on propagating parasites in laboratory snail colonies [53, 54]. However, infection rates were low, and many parasites died before shedding cercariae, the larval stage that infects humans. A safety and infectivity study using male parasites determined that a dose of 30 cercariae resulted in good infection rates but carried a risk of acute schistosomiasis syndrome. The standard dose now used is 20 cercariae, associated with an infection rate of approximately 80%. Similar studies were carried out with female cercariae, leading to the adoption of the same dose. Several factors have been studied, including the expression of key antigens used in diagnostics [circulating anodic antigen (CAA) and circulating cathodic antigen (CCA)], cytokine production, cell-mediated immunity, and microbial changes.

This model has recently been transferred to Uganda (CHI-S-Uganda) to gather more data on host–parasite interactions and correlates of protection in endemic settings [55]. The study focuses on a high-exposure fishing community and a minimal-exposure university community. Approximately 66 volunteers will be challenged with single-sex cercariae, with the initial number set at 10, and potentially increasing to 20 if deemed safe. Volunteers from the fishing community will be given praziquantel to eliminate any existing infection. To produce the challenge agent, a laboratory colony of local snails was created to propagate parasites.

HIC studies with *S. mansoni* have been crucial in identifying diagnostic targets. Antibody detection alone cannot differentiate between past and current infections, so challenge models provide a unique opportunity to identify accurate targets that are low at baseline and increase after infection. Primary infection markers, such as anti-CAA IgM and IgG antibodies, have been identified (in pre-print [56]). The development of an antibody-detection tool based on these findings would have implications for travel medicine and surveillance in post-elimination and emerging transmission zones.

## Other perspectives on HIC studies

### Pandemic response and preparedness—the role for human challenge

A clinical consortium, facilitated through the HIC-Vac network and other contacts, was formed to design and conduct SARS-CoV-2 challenge studies. The WHO acknowledged the importance of these studies and issued two guidance documents outlining feasibility and ethical considerations [57, 58]. These studies required strong scientific justifications and a thorough analysis of participant risks. The unique data obtained from challenge studies, including infection kinetics, correlates of protection, and vaccine testing, as well as their potential policy implications were key justifications. Participant safety remained the top priority.

Initially, there was reluctance within the UK Government to consider HIC studies at the start of the pandemic, but influential figures eventually provided strong support. Nevertheless, it is crucial to prepare in advance for future pandemics by establishing criteria for justified challenge studies and decision-making procedures, conducting ethical approvals, manufacturing under Good Manufacturing Practices, and engaging the public. Adequate preparation can enhance responsiveness, even though the speed at which pandemics evolve remains a challenge. Additionally, addressing challenges related to sample and data sharing is essential, as future pandemics may have less existing information on the causative agent.

## HICs—industry perspectives

HIC studies offer unique advantages to small biotechnology firms and pharmaceutical companies. Large pharmaceutical companies focus on internal decision-making, while small Biotech’s aim to gain rapid evidence of efficacy for future funding and company value. Generating data quickly through challenge studies benefits Biotech survival and provides proof-of-concept efficacy data. Challenge studies can optimise dosing, select candidates, and de-risk phase II studies. Developers must consider that regulators prioritise field efficacy data for initial licensing, but study failures can damage a company’s credibility and future investment prospects.

In the context of testing novel COVID-19 vaccines, large-scale placebo-controlled trials are no longer ethical or feasible. Challenge studies serve as a faster, cost-effective alternative for gathering data on specific SARS-CoV-2 strains. However, limitations include the need for constant re-evaluation and ensuring real-world scenarios, particularly as people became infected and/or vaccinated as the pandemic progressed. Challenge studies may provide a false representation of real-life variance, impacting the power of subsequent phase II/III trials. Other limitations include differences in participants compared to the target population, inability to simulate severe disease, and potential discrepancies in exposure routes and infective doses, leading to false indications of vaccine efficacy.

While HIC studies can be valuable for deriving correlates of protection, industry stakeholders may hesitate to fund such studies. Collaborations with academic partners can help integrate exploratory endpoints into industry challenge studies. The decision to use HIC studies is complex and depends on various factors. They play a vital role in de-risking by demonstrating proof of concept, providing comparisons with standard care, evaluating vaccines and antigens, and influencing licensing decisions for vaccines for those travelling to the countries where the diseases are endemic.

## Public and patient involvement and engagement

HIC studies raise social and ethical issues and require input from social scientists. Whilst participants are motivated by financial compensation, healthcare benefits, and community health contributions, understanding the study and trust in the institution and research review systems are crucial. Participants experience burdens such as infection symptoms and regular blood draws, limited right to movement while in-residence, anxiety around potential long-term effects of deliberate infection, and anxieties about early study exit. Although monetary compensation often results in an increase in interest in participating, it does not appear to cloud the understanding of the risks associated with participating. These were all main findings from embedded social science and empirical ethics research done as part of the CHMI-SIKA study [59–61].

The ‘reshape–seed’ study (Oxford University Clinical Research Unit, Vietnam) identified motivations for participating including potential health benefits, considerations of safety, ethical approval, and institute reputation. Previous experience with the disease, positive outcomes in previous studies, and expert advice increase the likelihood of participation. Conversely, parental disapproval, family emergencies, and damaging rumours decrease participation.

First-hand accounts from previous HIC study volunteers highlight the importance of providing comprehensive information on the risks involved. Informed consent should cover long-term impacts, risks, and specific procedures like X-rays and therapies. Despite potential drawbacks, volunteers believe that the benefits outweigh the risks. The volunteers also mentioned sharing study results with participants can foster a sense of belonging and maintains interest in challenge studies.

Early stakeholder engagement is crucial for designing acceptable HIC studies. To normalise challenge studies in certain settings, particular strategies have resulted in practical changes. This has been done extensively in Kenya through the Kenya Medical Research Institute (KEMRI). Participants are shown the volume of blood collected and compensated at regular intervals (e.g. weekly) throughout the study period, rather than receiving a lump sum at the end. Community involvement at the grant development stage has ensured views and concerns of study communities are carefully considered in the development and planning of HIC studies. Lessons learned from social science research, and engagement with stakeholders and the public around HIC studies have substantially contributed to WHO’s guidance on the ethical conduct of HIC studies. Future initiatives aim to involve volunteers in the co-development of study information materials and videos for engagement with key stakeholders, as well as explore the diverse attitudes toward HIC studies in different settings.

## Conclusions from the meeting

The meeting provided a platform to share the latest advances in human challenge studies globally. The attendees highlighted the unique international diversity of the HIC-Vac consortium and its ability to engage those from developing countries. It also highlighted the importance of the collaborations between academic and clinical institutions, particularly between the UK and LMICs. These collaborations drive the success of HIC studies in accelerating vaccine discovery and rollout. Presenters emphasized the crucial role of HIC studies in unravelling the mechanisms of pathogen invasion, understanding immune responses to infection, and assessing the effectiveness of vaccines and therapeutics against infectious diseases, while underscoring the importance of public and patient engagement and the role of industry. The meeting fostered a vigorous collaborative spirit, encouraging networking and facilitating discussions on novel ideas and potential studies to advance vaccine development for infections of high global impact. Highlighting the advantages and limitations of human challenge studies, this meeting concluded that experimental infection plays an increasing tole in the range of studies to accelerate vaccine development.

## Data Availability

No data are associated with this article.
